# Distinct Gut Microbiota Profiles in Normal Weight Obesity and Their Association With Cardiometabolic Diseases: Results From Two Independent Cohort Studies

**DOI:** 10.1002/jcsm.13644

**Published:** 2024-12-26

**Authors:** Wenjie Wang, Feijie Wang, Yihan Li, Yuwei Shi, Xiaoyan Wang, Xinyu Chen, Weifang Zheng, Julianna C. Hsing, Ying Lu, Yi‐Shuan Wu, Ann W. Hsing, Juntao Kan, Wei He, Shankuan Zhu

**Affiliations:** ^1^ Chronic Disease Research Institute, the Children's Hospital, and National Clinical Research Center for Child Health, School of Public Health, School of Medicine Zhejiang University Hangzhou China; ^2^ Department of Nutrition and Food Hygiene, School of Public Health, School of Medicine Zhejiang University Hangzhou China; ^3^ Nutrilite Health Institute Shanghai China; ^4^ Lanxi Hospital of Traditional Chinese Medicine, Lanxi Zhejiang China; ^5^ Department of Epidemiology and Population Health, Stanford School of Medicine Stanford University Stanford California USA; ^6^ Department of Biomedical Sciences, Stanford School of Medicine Stanford University Stanford California USA; ^7^ Department of Medicine, Stanford Prevention Research Center, Stanford School of Medicine Stanford University Stanford California USA

**Keywords:** Cardiometabolic diseases, Gut microbiota, Independent cohort, Normal weight obesity

## Abstract

**Background:**

Normal weight obesity (NWO) is characterized by excess body fat in individuals with normal body mass index (BMI). This study aimed to investigate gut microbiota alterations in NWO and their potential associations with cardiometabolic diseases (CMD) risk in two independent cohorts.

**Methods:**

Our NWO‐CMD mortality analysis included 168 099 adults with normal BMI from two large open‐access databases, while our NWO‐gut microbiota study involved 5467 adults with normal BMI from two independent cohorts: the WELL‐China cohort and the Lanxi cohort. NWO was defined as having a normal BMI (18.5–23.9 kg/m^2^) but an excess per cent body fat (PBF, ≥ 25% in men and ≥ 35% in women). Normal weight lean was defined as having a normal BMI and normal PBF. The 16S rRNA gene sequencing method was used to analyse gut microbiota data.

**Results:**

The study comprised 3620 (64.0% female, median age 58 years) and 1847 (64.3% female, median age 56 years) participants from the WELL‐China and Lanxi cohorts. In our meta‐analysis, NWO is associated with 26% (95% CI: 1.07–1.41) higher risk of CMD mortality. Gut microbial analyses indicated that the NWO group exhibited reduced levels of observed species (*p* = 0.009 and *p* = 0.013) and Chao 1 index (*p* = 0.002 and *p* = 0.002) and altered gut microbial compositions (*p* = 0.009 and *p* < 0.001) compared with the NWL group. Seven genera were consistently observed to be associated with NWO in both two cohorts (all Q < 0.25). Among them, five (*Fusobacterium*, *Ruminococcus gnavus group*, *Ruminococcus torques group*, *Coprococcus* and *Christensenellaceae_R7_group*) have been previously linked to obesity, while the other two (*Phascolarctobacterium* and *Clostridia_UCG‐014*) were minimally reported. We also found statistically significant differences in the microbial composition between the NWO group and the obesity group (*p* = 0.001 and *p* = 0.001). Furthermore, the NWO‐related gut microbiome was associated with an elevated risk of hypertension, dyslipidaemia and metabolic syndrome, the corresponding HR (95% CIs) were 1.11 (1.01–1.22), 1.19 (1.10–1.29) and 1.17 (1.05–1.30) in the WELL‐China cohort and 1.14 (1.02–1.27), 1.15 (1.02–1.29) and 1.16 (1.02–1.32) in the Lanxi cohort.

**Conclusions:**

These two large cohorts provided reliable evidence that gut microbiota alterations in NWO resemble those found in obesity, yet also display unique aspects. This distinct microbiota profile may contribute to heightened cardiometabolic risks in adults with normal BMI.

## 1 Introduction

The gut microbiome is a pivotal environmental factor in the development of obesity, as evidenced by bacterial transplantation experiments in rodents [1]. Studies in humans have also linked gut microbiota to obesity as defined by body mass index (BMI), revealing microbial dysbiosis among individuals with obesity [2, 3]. In such studies, normal BMI was generally considered to represent the control or healthy group. However, it is noteworthy that BMI may not accurately distinguish fat content and lean mass [4], leading to instances of obesity within the normal BMI range.

Normal weight obesity (NWO), characterized by an elevated per cent body fat (PBF) despite a normal BMI, represents a distinct obesity phenotype [5]. Individuals with NWO often escape detection in public health screenings owing to their normal BMI. However, NWO represents a public health concern, that is estimated to affect ~ 4.5%–22% of the global population [6]. Until now, limited evidence exists regarding whether gut microbiota is altered in individuals with NWO. One study investigated gut microbiota features in 32 haemodialysis patients with NWO, revealing statistically significant reductions in α‐diversity and abundances of butyrate‐producing bacteria [7]. Nevertheless, this study's applicability to the general population was constrained by its exclusive focus on patients undergoing haemodialysis.

Although several studies have reported a heightened risk of cardiometabolic diseases (CMD) in individuals with NWO, [8, 9] evidence regarding the relationship between NWO and CMD mortality is limited and conflicting [10, 11]. One prospective study of 1528 subjects aged ≥ 60 years found that NWO was not associated with CMD mortality [10], while another study involving 6171 adults showed a marginally positive association of NWO with CMD mortality in women but not in men [11]. Furthermore, evidence has suggested that disruptions of the intestinal micro‐ecological equilibrium play a role in the pathogenesis of CMD, potentially impacting metabolic health [12, 13]. Despite these findings, the connection between NWO‐related changes in gut microbiota and cardiometabolic diseases remains inadequately explored.

This study aimed to address these gaps using microbiome data from two independent cohorts–the Wellness Living Laboratory (WELL)‐China cohort and the Lanxi cohort. The primary objectives were to investigate whether (1) NWO is associated with alteration in gut microbiota compositions and (2) NWO‐related gut microbiome changes are associated with CMD. As a secondary objective, we also conducted a meta‐analysis of the association of NWO with CMD mortality using data from two large open‐access databases.

## 2 Materials and Methods

### 2.1 Study Design and Population

The NWO‐mortality analyses utilized data from two large prospective cohorts: the US National Health and Nutrition Examination Survey NHANES) and the UK Biobank. Both cohorts were approved by institutional review boards and all participants provided informed consent. Further detailed information about the US NHANES and UK Biobank studies was described in the Supplementary methods.

The NWO‐gut microbiota analyses utilized data from two independent cohorts: the WELL‐China cohort and the Lanxi cohort. WELL‐China is a population‐based cohort study conducted in three districts of Hangzhou, China. The study design and sample collection details have been previously reported [14]. Briefly, the study recruited 10 268 participants aged 18–80 years, who were long‐term residents of the selected district, via random and quota‐based sampling. The Lanxi cohort, on the other hand, is a community‐based study conducted in Lanxi, Zhejiang Province, China. Procedures related to sample collection, questionnaire surveys and anthropometric measurements have been published previously [15]. In summary, the cohort recruited a total of 4503 participants aged 18–80 years between 2017 and 2019, including 1805 rural and 2698 urban residents.

The flow chart of the participant selection for gut microbiota analyses was presented in Figure S1. Exclusions were made for participants with a BMI < 18.5 kg/m^2^, those classified as overweight or obese (BMI ≥ 24 kg/m^2^), individuals with missing data on anthropometric measurements, dual‐energy X‐ray absorptiometry (DXA) scans and gut microbiota information, as well as those who self‐reported baseline gastrointestinal diseases and cancer. As a result, the analysis included a total of 3620 participants from the WELL‐China cohort and 1847 participants from the Lanxi cohort, all of whom were within the normal weight range (18.5 kg/m^2^ ≤ BMI < 24 kg/m^2^).

The WELL‐China cohort study protocol received approval from the Institutional Review Boards of Zhejiang University, China (No. ZGL201507‐3) and Stanford University, USA (IRB‐35020). The Lanxi Cohort study protocol obtained approval from the Ethics Committee of the School of Public Health, Zhejiang University (No: ZGL2012‐12), China. Written informed consent was obtained from all study participants.

### 2.2 Definition of Normal Weight Obesity (NWO)

In the WELL‐China and Lanxi cohorts, normal weight obesity (NWO) was defined as participants with normal BMI (18.5–23.9 kg/m^2^), but excess per cent body fat (≥ 25% in men and ≥ 35% in women) [16]. Normal weight lean (NWL) was defined as participants with normal BMI and normal per cent body fat. Whole‐body DXA scans (software version 11.40.004, GE Lunar Prodigy; GE Healthcare, Milwaukie, WI, USA) were performed in all participants to measure total and regional body fat mass in both cohorts. DXA operates by using two X‐ray beams of different energy levels. As these beams pass through the body, detectors measure the amount of X‐ray radiation absorbed by various tissues. These data are then used to generate detailed images and precise measurements of bone density and body composition. Compared with bioelectrical impedance analysis (BIA), DXA offers more accurate assessments of bone density and body composition.

### 2.3 Definition of Normal Weight Obesity (NWO)‐Microbial Index

Details on faecal sample collection, DNA extraction and 16S rRNA sequencing are provided in Data S1. To summarize the NWO‐related gut microbial genera, we developed the NWO‐microbial index (MI). This index was constructed based on the genera associated with NWO in the two cohorts, following the method outlined by Jiang et al. in a previous study [17]. The microbial index was calculated using the following formula:

$I^{P}=\sum_{j=1}^{n}G_{\mathrm{ij}}$ $\;I^{N}=\sum_{j=1}^{m}G_{\mathrm{ij}}$ $X_{\mathrm{MI}}=\frac{I^{P}}{n}-\frac{I^{N}}{m}$

$${\text{Standardized}}_{\mathrm{MI}}=\frac{X-\bar{X}}{\mathrm{SD}}$$

where $G_{\mathrm{ij}}$ represents the relative abundance of the genus j, i is the number of individuals. *P* is a subset of all NWO‐positive correlated genera, *N* is a subset of all NWO‐negative correlated genera, n is the number of genera positively associated with NWO, m is the number of genera negatively associated with NWO, $\bar{X}$ is the mean value of X_*MI*_, and SD is the standard deviation of X_*MI*_. This approach allows us to comprehensively assess the microbial genera associated with NWO, providing a standardized measure for comparison across cohorts.

### 2.4 Definition of Cardiometabolic Diseases

In the WELL‐China and Lanxi cohorts, hypertension was defined as systolic blood pressure (SBP) of ≥ 140 mmHg or diastolic blood pressure (DBP) of ≥ 90 mmHg or the currently use of antihypertensive drugs. Diabetes was defined as fasting blood glucose levels ≥ 7.0 mmol/L or haemoglobin A1C (HbA1c) levels ≥ 6.5%, or a self‐reported history of diabetes‐related medication use. Dyslipidaemia was defined as total cholesterol (TC) ≥ 6.2 mmol/L, triglycerides (TG) ≥ 2.3 mmol/L or low‐density lipoprotein cholesterol (LDL‐C) ≥ 4.1 mmol/L or high‐density lipoprotein cholesterol (HDL‐C) < 1.0 mmol/L, or the self‐reported using lipid‐lowering medications. Metabolic syndrome (MetS) was defined in accordance with the revised criteria of the International Diabetes Federation (IDF).

### 2.5 Definition of Covariates

In the WELL‐China and Lanxi cohorts, a face‐to‐face questionnaire survey was utilized to gather information regarding age, sex, smoking status (never smoker, ever smoker or current smoker), drinking status (non‐drinker, occasional drinker and frequent drinker), marital status (unmarried, married or other), physical activity (inactive, insufficiently active and active), educational level (elementary school or less, middle school, high school and college or above) and annual income (< 50 000￥, 50 000–100 000￥and > 100 000￥), dietary total energy intake (kcal/day) and antibiotic use (yes, no). In the Lanxi cohort, we additionally adjusted for region (urban and rural). Detailed information regarding our assessment of lifestyle and diet is provided in Data S1.

### 2.6 Statistical Analysis

In the NWO‐gut microbiota analyses, continuous and categorical variables are presented as means (standard deviations, SDs) and numbers (percentages), respectively. We compared baseline characteristics between the NWO and NWL groups using the Student's *t*‐test and chi‐squared test.

We employed a linear regression model to assess the differences in gut microbiome α‐diversity indexes between the NWO and NWL groups. Additionally, we utilized principal coordinate analysis (PCoA) and permutational multivariate analysis of variance (PER‐MANOVA) based on the Bray–Curtis distance to assess gut microbial β‐diversity dissimilarities between the NWO and NWL groups.

Based on metagenomes inferred from 16S rRNA data, we predicted the functional pathways of gut microbiota from the MetaCyc metabolic pathway database using PICRUSt2. Microbiome multivariable associations with linear model (MaAsLin2) were applied to identify the gut microbial genera and functional pathways associated with NWO. The Benjamini–Hochberg method was used to control the false discovery rate (FDR), with a Q value (FDR *P*) < 0.25 being considered statistically significant. Our analyses included only microbial genera with a mean relative abundance > 0.05% and that appeared in at least 10% of the samples. To identify NWO‐related metabolic mechanisms, we used previously measured metabolomics data from 137 participants with normal BMI in the WELL‐China cohort. The differential metabolites were tested using a linear regression model. Functional enrichment was performed using the pathway analysis features in MetaboAnalyst 6.0. Details regarding metabolomic assays has been reported previously [18].

Subsequently, Spearman correlation analysis was performed to explore the association between these NWO‐related gut microbial genera and 11 cardiometabolic risk indicators. We performed two‐sample mendelian randomization (MR) analysis to investigate the causal relationship between NWO‐related microbiota features and CMD risk indicators. The random‐effects inverse‐variance weighted (IVW) method is reported to be slightly more powerful than the others under certain conditions, therefore, the results with more than one IV were mainly based on the IVW method. The MR analysis was performed using the ‘*TwoSampleMR*’ package.

Multivariate linear models were used to assess the association of NWO‐MI—an index summarizing NWO‐related microbial genera—with per cent body fat, per cent android fat and per cent gynoid fat. Multivariable logistic regression models were used to investigate the association between NWO‐MI and CMD, including hypertension, diabetes, dyslipidaemia and metabolic syndrome. We used a random‐effects meta‐analysis to pool estimates from the two cohorts.

Spearman's partial correlations were used to explore the associations of NWO‐related gut microbial genera with diet and other lifestyle factors, adjusted for age and sex. All multivariate analyses were adjusted for age, sex, marriage, smoking, drinking, physical activity, education, income, total energy intake and antibiotic use unless otherwise indicated. Multiple imputation methods were used to impute covariates with missing values. All analyses were performed separately in the WELL‐China and Lanxi cohorts.

In the NWO‐CMD mortality analyses, we utilized Cox proportional hazards regression models. The proportional hazards assumption was tested using Schoenfeld residuals. Follow‐up times were calculated from baseline until the date of death or end of follow‐up, whichever occurred first. For the meta‐analysis, we used *I* ^2^ statistics to assess the heterogeneity across cohorts, a fixed‐effect model was used to combine the effect sizes when *I* ^2^ < 50%, otherwise, a random‐effect model was employed. All statistical analyses were performed in R version 4.2.1, and a two‐sided *P* value < 0.05 was considered statistically significant unless otherwise stated.

## 3 Results

### 3.1 NWO and Cardiometabolic Diseases Mortality

**FIGURE 1:**
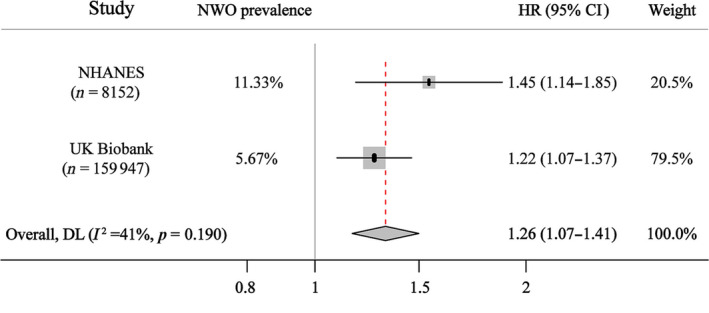
NWO and cardiometabolic mortality in US NHANES and UK Biobank. HR (95% CIs) were calculated from Cox proportional hazard models, adjusted for age, sex, race, smoking status, drinking status, physical activity, education attainment and income level. The effect estimates from two cohorts were pooled using random effects meta‐analysis. NHANES, National Health and Nutrition Examination Study; NWO, normal weight obesity. Weight refers to the relative importance of each individual study to the overall results of the meta‐analysis.

We included 8152 and 159 947 adults with normal BMI from the US National Health and Nutrition Examination Survey (NHANES) and UK Biobank databases, respectively. During a median follow‐up period of 12.1 and 12.7 years, a total of 349 and 1570 CMD mortality were recorded in these two cohorts. Figure 1 shows the association between NWO and CMD mortality. NWO was associated with an increased risk of CMD mortality in the US NHANES and the UK Biobank, the corresponding HR (95% CIs) were 1.45 (1.14–1.85) and 1.22 (1.07–1.37), respectively.

### 3.2 NWO‐Related Gut Microbiota Alterations

**FIGURE 2:**
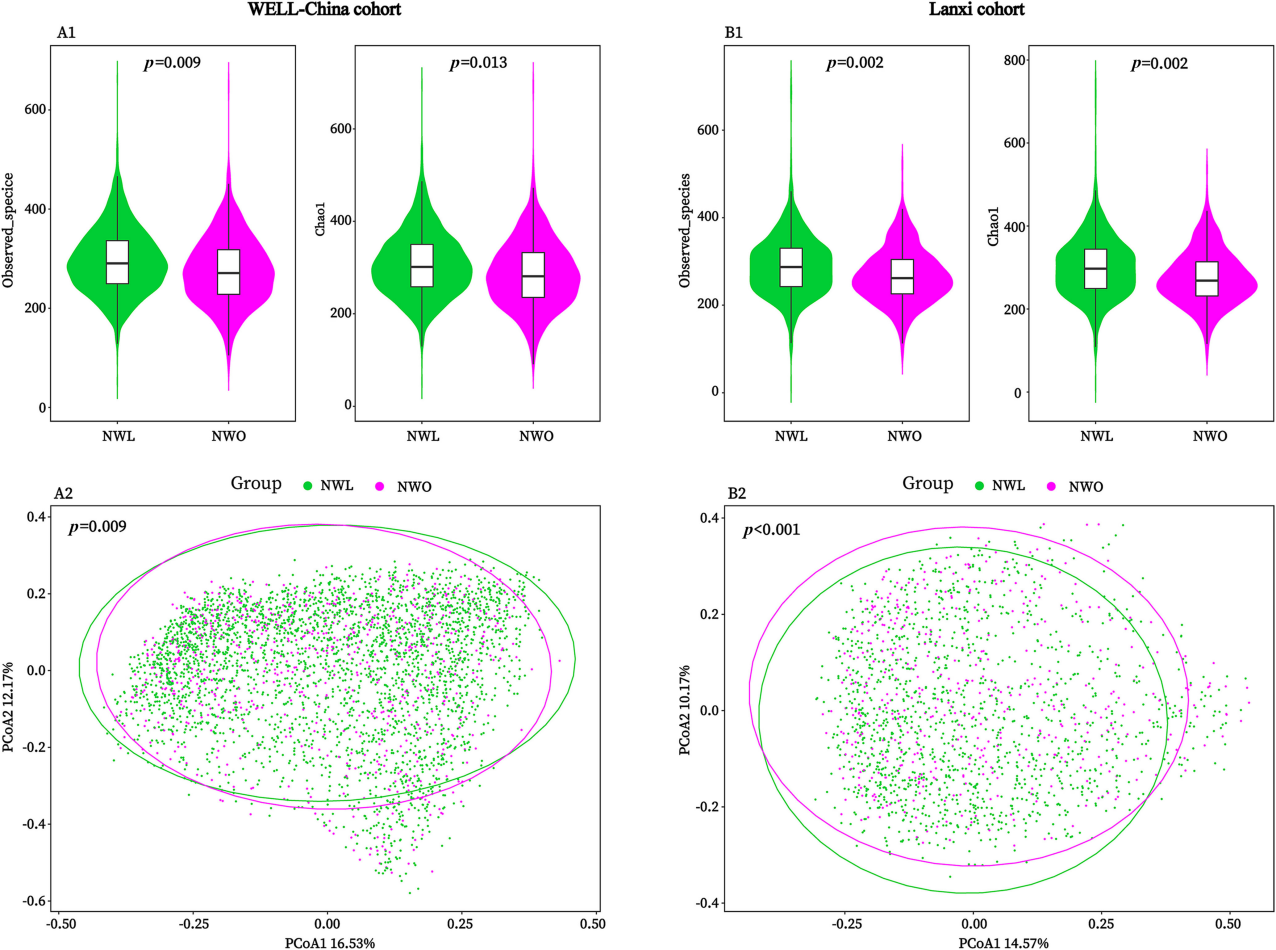
NWO‐related gut microbial α‐ and β‐diversity alterations in the WELL‐China (A1 and A2) and Lanxi cohorts (B1 and B2). *P* value for α‐diversity was calculated from the multivariable linear regression model, and adjusted for potential confounding factors (described in the text). Permutational ANOVA (999 permutations) was used to evaluate the *P* value for β‐diversity. NWL, normal weight lean; NWO, normal weight obesity.

The basic characteristics of the participants from the WELL‐China and Lanxi cohorts are presented in Table 1. In both cohorts, the NWO group exhibited a statistically significant reduction in faecal microbial richness, as indicated by a decrease in the α‐diversity parameters: observed species (*p* = 0.009 and 0.002) and Chao 1 index (*p* = 0.013 and 0.002) (Figure 2, A1–A2). Furthermore, both cohorts demonstrated a notable shift in gut microbial composition (β‐diversity) between the NWO and NWL groups (*p* = 0.009 and < 0.001) (Figure 2, B1–B2).

**TABLE 1:** Baseline characteristics of the study population in the Wellness Living Laboratory (WELL)‐China cohort and Lanxi cohort, respectively.^a^

	WELL‐China cohort			Lanxi cohort		
Characteristics	NWL	NWO	*p*	NWL	NWO	*p*
*N*	2885	735		1357	511	
Age (year)	55.81 (12.75)	56.43 (12.89)	0.232	56.29 (11.61)	56.20 (11.65)	0.881
Gender, %			0.789			0.066
Male	1034 (35.8)	268 (36.3)		496 (37.1)	164 (32.5)	
Female	1851 (64.2)	467 (63.7)		849 (62.9)	338 (67.5)	
Marriage status, %			0.057			0.064
Single	118 (4.1)	37 (5.0)		29 (2.2)	8 (1.6)	
Married	2483 (86.1)	630 (85.7)		1242 (92.3)	458 (91.2)	
Others	284 (9.8)	68 (9.3)		74 (5.5)	36 (7.2)	
Smoking status, %			0.009			0.019
Current smoker	509 (17.6)	97 (13.2)		202 (15.0)	57 (11.3)	
Ever smoker	192 (6.7)	51 (6.9)		72 (5.3)	34 (6.8)	
Never smoker	2184 (75.7)	587 (79.9)		1071 (79.6)	411 (81.9)	
Drinking status, %^b^			0.007			< 0.001
Nondrinker	1649 (57.2)	461 (62.7)		776 (57.7)	342 (68.1)	
Occasional drinker	691 (23.9)	168 (22.8)		315 (23.4)	100 (19.9)	
Frequent drinker	545 (18.9)	106 (14.4)		254 (18.9)	60 (12.0)	
Physical activity, %			0.018			< 0.001
Inactive	1153 (40.0)	324 (44.1)		799 (59.4)	347 (69.1)	
Insufficiently active	645 (22.3)	169 (23.0)		126 (9.4)	49 (9.6)	
Active	1087 (37.7)	242 (32.9)		420 (31.2)	106 (21.3)	
Education level, %			0.011			< 0.001
Elementary school or less	592 (20.5)	119 (16.2)		570 (42.4)	160 (31.9)	
Middle school	964 (33.4)	246 (33.5)		441 (32.8)	175 (34.9)	
High school	691 (23.9)	198 (26.9)		199 (14.8)	102 (20.3)	
College or above	638 (22.1)	172 (23.4)		135 (10.0)	65 (12.9)	
Annual income level, CNY			0.040			< 0.001
< 50 000￥	1924 (66.7)	464 (63.1)		630 (46.8)	172 (34.3)	
50 000–11 000￥	809 (28.0)	233 (31.7)		373 (27.7)	167 (33.3)	
> 11 000￥	152 (5.3)	38 (5.2)		341 (25.4)	163 (32.3)	
Total energy intake (kcal/day)	1726.04 (682.73)	1756.73 (720.81)	0.298	2150.18 (735.77)	2201.43 (741.58)	0.186
Region, %						< 0.001
Rural	—	—	—	636 (47.4)	130 (25.8)	
Urban	—	—	—	709 (52.6)	372 (74.2)	

^a^ Data are presented as mean with standard deviation (SD) for continuous variables and *n* (%) for categorical variables.

^b^ Drinking status: occasional drinker/frequent drinker was defined as whether drinks more than 12 times per year.

In the WELL‐China cohort, we identified nine microbial genera that were associated with NWO. In the Lanxi cohort, NWO was found to be significantly associated with 14 individual genera. Seven differential genera—*Clostridia_UCG‐014*, C*hristensenellaceae_R7_group*, *Coprococcus*, *Fusobacterium*, *Ruminococcus gnavus group*, *Phascolarctobacterium* and *Ruminococcus torques group*—overlapped between the two cohorts. This overlap accounted for 77.8% and 50% of the differential genera in the WELL‐China and Lanxi cohorts, respectively (Table 2).

**TABLE 2:** Association of gut microbial genus with normal weight obesity (NWO) from the MaAsLin2 multivariate adjusted model.

WELL‐China cohort^a^				Lanxi cohort^b^			
Genus	Coefficient	*p*	Q value^c^	Genus	Coefficient	*p*	Q value^c^
Enriched in the NWO group							
***Fusobacterium***	0.118	0.041	0.223	***Fusobacterium***	0.358	< 0.001	< 0.001
***Ruminococcus torques***	0.089	0.011	0.067	***Ruminococcus torques***	0.098	0.003	0.019
***Ruminococcus gnavus***	0.086	0.034	0.209	***Ruminococcus gnavus***	0.270	< 0.001	< 0.001
***Phascolarctobacterium***	0.089	0.046	0.235	***Phascolarctobacterium***	0.211	0.003	0.015
—	—	—	—	*Megamonas*	0.242	0.002	0.015
—	—	—	—	*Lachnoclostridium*	0.213	< 0.001	< 0.001
Depleted in the NWO group							
***Clostridia_UCG‐014***	−0.236	< 0.001	0.009	***Clostridia_UCG‐014***	−0.342	< 0.001	0.004
***Christensenellaceae_R7_group***	−0.121	0.024	0.154	***Christensenellaceae_R7_group***	−0.273	0.001	0.009
***Coprococcus***	−0.091	0.049	0.242	***Coprococcus***	−0.123	0.045	0.238
*Prevotella*	−0.137	0.006	0.045	*Holdemanella*	−0.502	< 0.001	< 0.001
*Muribaculaceae*	−0.129	0.040	0.221	*UCG.002*	−0.272	< 0.001	0.002
—	—	—	—	*Akkermansia*	−0.208	0.033	0.122
—	—	—	—	*Dorea*	−0.115	0.026	0.104
—	—	—	—	*Bacteroides*	0.088	0.006	0.032

*Note:* Bold text represents overlapping differential gut microbiota in the WELL China cohort and the Lanxi cohort.

Abbreviation: MaAsLin2, microbiome multivariable associations with linear model.

^a^ Adjusted for age, sex, marriage, smoking, drinking, physical activity, education, income, total energy intake and antibiotic use in the WELL‐China cohort.

^b^ Additionally adjusted for region (rural and urban) in the Lanxi cohort.

^c^ The Q values were calculated using the Benjamini–Hochberg method with Q value < 0.25 was considered statistically significant.

### 3.3 NWO‐Related Microbial Functions and Metabolic Pathways

**FIGURE 3:**
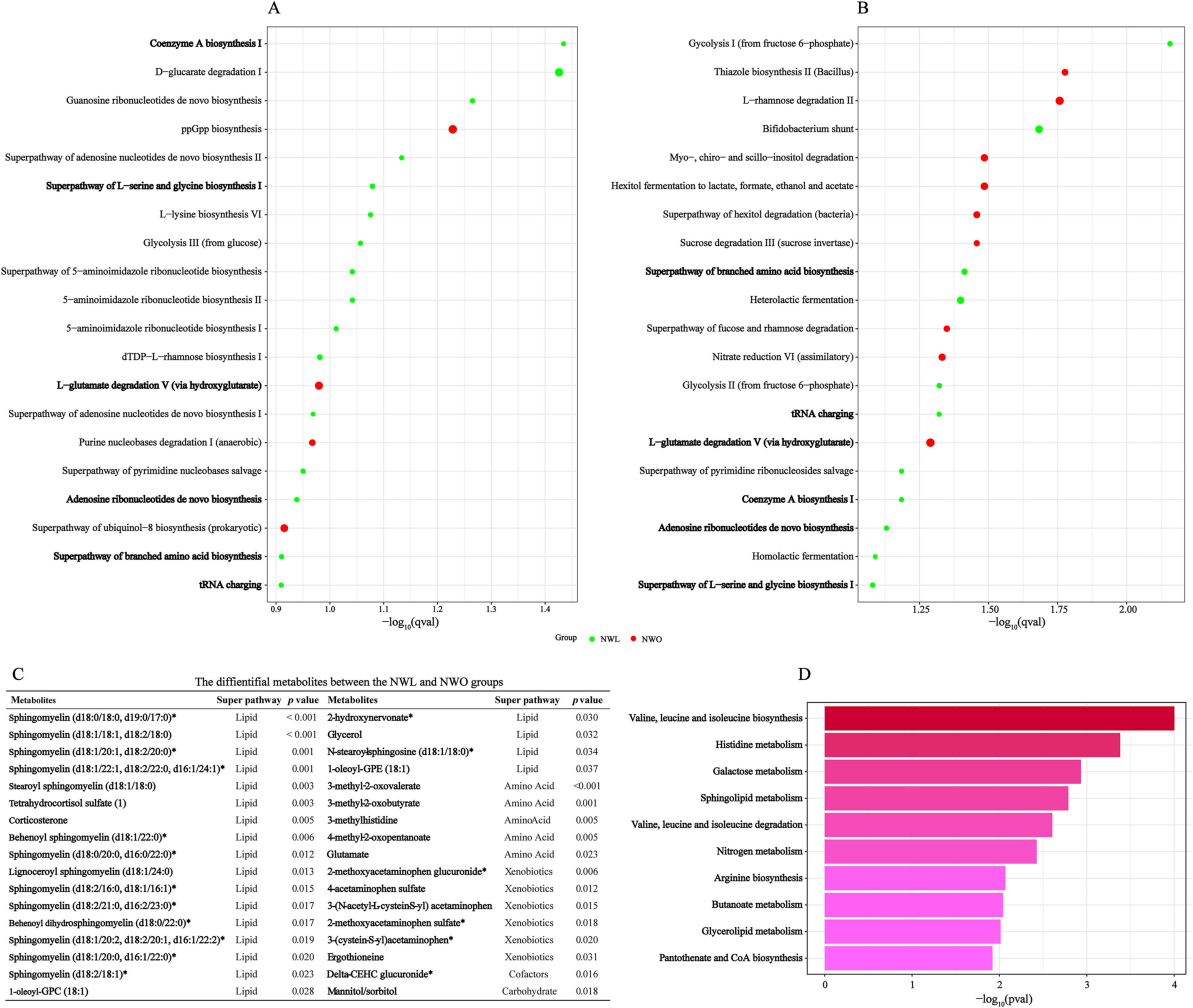
NWO‐related gut microbial function and metabolic pathways. Differences in the predicted microbial function between the NWL and NWO groups in the WELL‐China (A) and Lanxi cohorts (B). The differential metabolites between the NWL and NWO groups (C). The metabolic pathway analysis of differential metabolites between the NWL and NWO groups (D).

Figure 3 showed the predicted microbial functions in the WELL‐China and Lanxi cohorts. We found that six functional pathways overlapped in the two cohorts. Specifically, pathways related to amino acid degradation (L‐glutamate) were enriched in the NWO group, while pathways related to amino acid biosynthesis (branched amino acid, L‐serine and glycine), cofactor biosynthesis (coenzyme A) and nucleotide biosynthesis (adenosine ribonucleotides) were depleted in the NWO group (Figure 3A,B).

We identified 34 differential metabolites between the NWL and NWO groups. The most abundant metabolites were from the lipid super pathway, followed by amino acid, xenobiotics and cofactors super pathways. In pathway analysis, we found that these metabolites were enriched in 10 pathways. Among them, amino acid biosynthesis (valine, leucine and isoleucine), amino acid degradation (valine, leucine and isoleucine) and cofactor biosynthesis (pantothenate and coenzyme A) that were identified in microbial function also appear in metabolic pathways (Figure 3C,D).

### 3.4 NWO‐Related Microbial Genera and Cardiometabolic Risk Indicators

**FIGURE 4:**
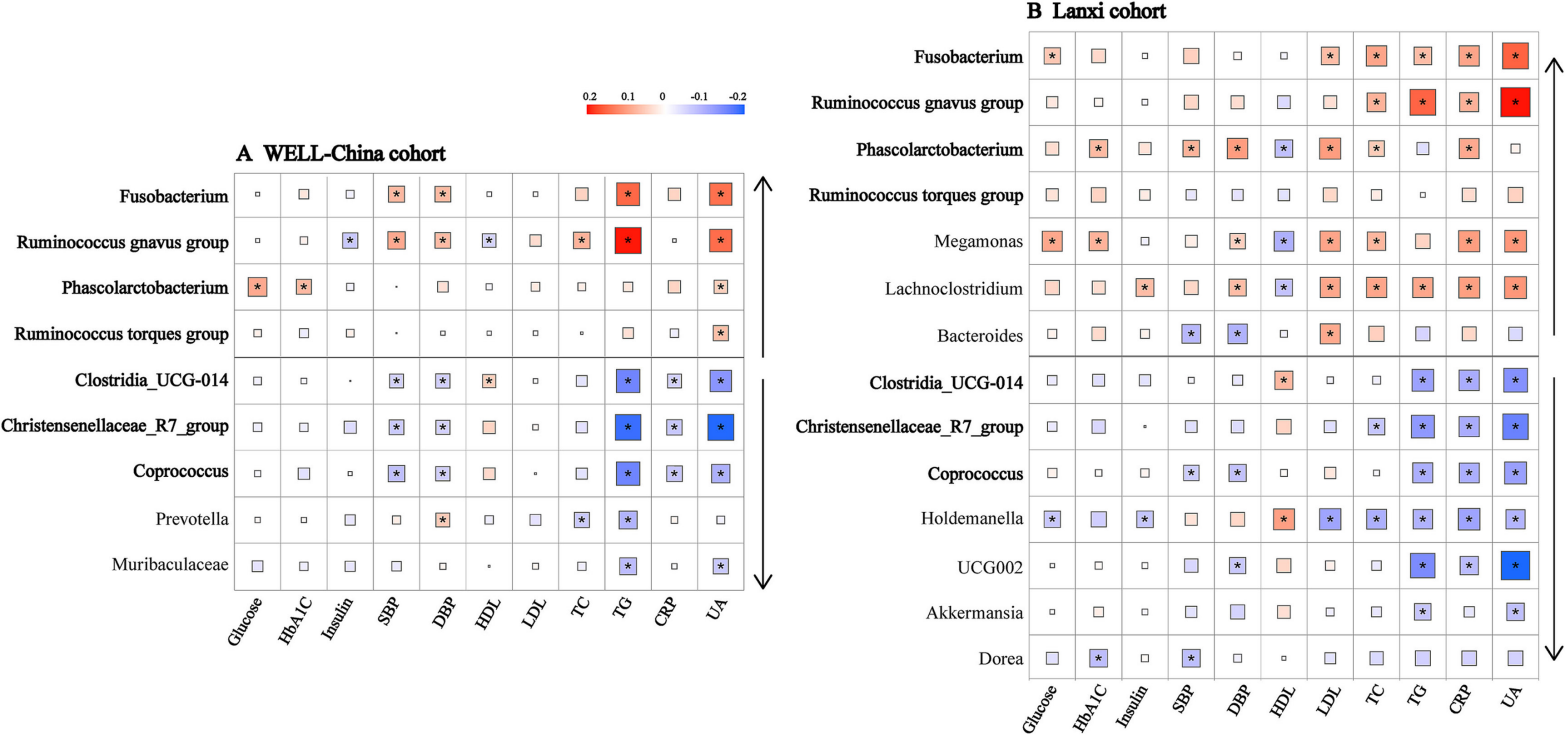
NWO‐related gut microbiota and cardiometabolic risk indicators in the WELL‐China (A1) and Lanxi cohorts (A2). *P* value was corrected using the Benjamini‐Hochberg false discovery rate (FDR). *FDR‐corrected *p* < 0.05. CRP, C‐reactive protein; DBP, diastolic blood pressure; HDL, high‐density lipoprotein; LDL, low‐density cholesterol; NWO, normal weight obesity; SBP, systolic blood pressure; TC, total cholesterol; TG, triglycerides; UA, uric acid. Upward arrows indicated the genera enriched in the NWO group, whereas downward arrows indicated the genera depleted in the NWO group.

In both the WELL‐cohort and Lanxi cohorts, we observed that the genera that were enriched in the NWO group, were positively correlated with most of the 11 investigated cardiometabolic risk indicators while the three genera that were depleted in the NWO group, were negatively correlated with cardiometabolic risk indicators (Figure 4A,B). Specifically, *Fusobacterium* and *Ruminococcus gnavus group* were correlated with higher levels of systolic blood pressure, diastolic blood pressure, triglyceride and uric acid, whereas *Coprococcus*, *Clostridia_UCG‐014* and *Christensenellaceae_R7_group* were correlated with the lower levels of triglyceride, C‐reactive protein and uric acid.

In the MR analysis, the genetically predicted relative abundance of *Fusobacterium* was associated with higher levels of systolic blood pressure, diastolic blood pressure and uric acid. In contrast, the genetically predicted *Coprococcus* abundance was associated with lower C‐reactive protein concentration (OR = 0.97, 95% CI = 0.95–0.99, *p* = 0.010) and higher HDL cholesterol level (OR = 1.02, 95% CI = 1.00–1.05, *p* = 0.038). In addition, the genus *Christensenellaceae_R7_group* was also causally associated with a higher level of LDL cholesterol (Table S1).

### 3.5 NWO‐Microbial Index and Cardiometabolic Diseases

**FIGURE 5:**
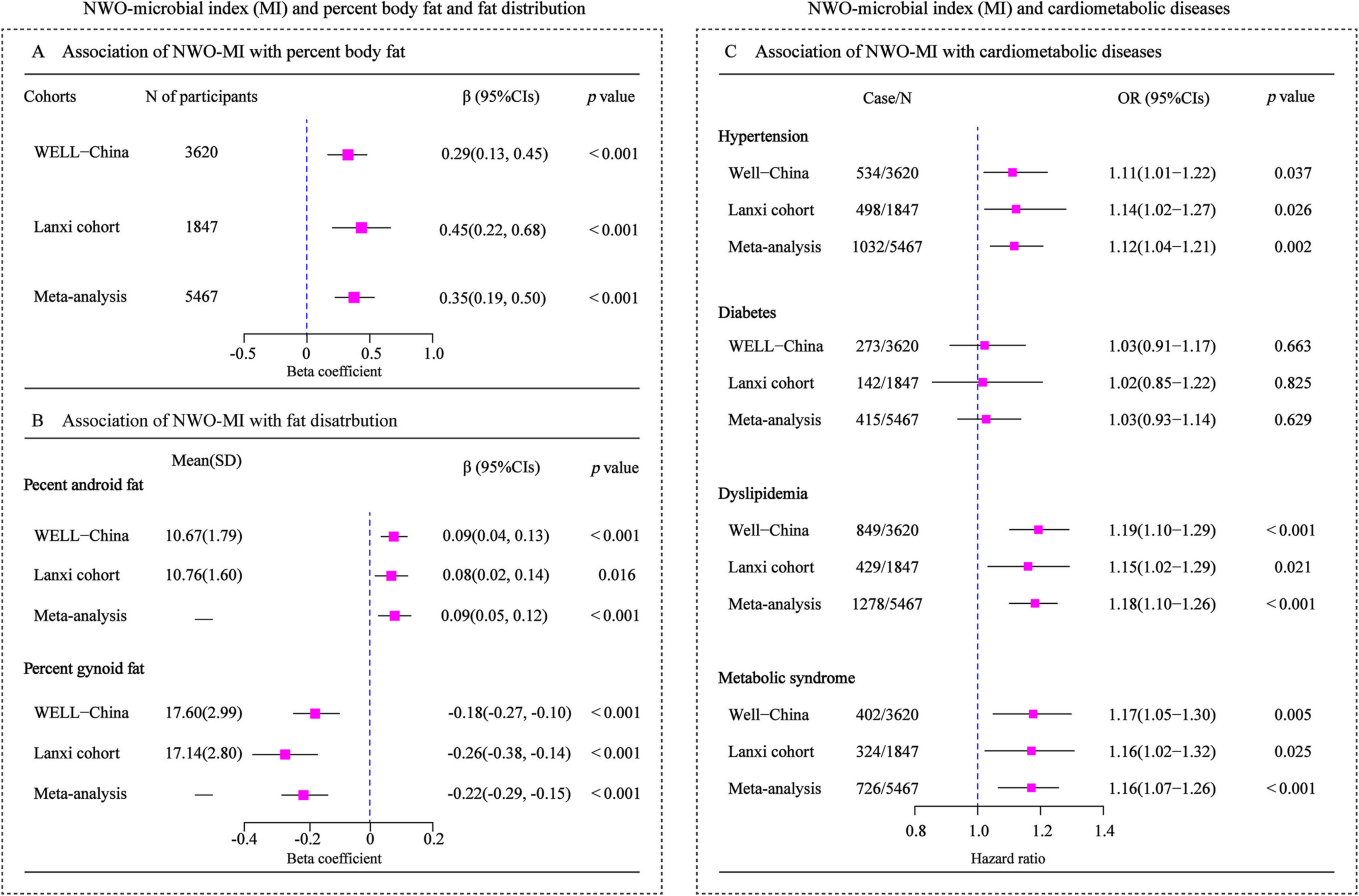
NWO‐microbial index and cardiometabolic diseases. Multivariable linear regression model was used to evaluate the association of NWO‐microbial index (MI) with percent body fat (A), and percent android/gynoid fat (B) in the WELL‐China cohort and Lanxi cohort. Multivariable logistic regression was used to estimate the association of NWO‐MI (per‐SD increase) with CMD risk in the WELL‐China cohort and Lanxi cohort (C). The effect estimates from two cohorts were pooled using random effects meta‐analysis. CMD, cardiometabolic diseases.

The NWO‐microbial index (MI), which summarized NWO‐related microbial genera, exhibited a positive association with per cent body fat and per cent android fat, whereas showing a negative association with per cent gynoid fat in both the WELL‐China and Lanxi cohorts (Figure 5A,B).

NWO‐MI was consistently significantly associated with an elevated risk of CMD in the WELL‐China and Lanxi cohorts. For each standard deviation increment in the NWO‐MI, there was an 11% and 14% increased risk of hypertension (95% CI: 1.01–1.22 and 1.02–1.27), a 19% and 15% increased risk of dyslipidaemia (95% CI: 1.10–1.29 and 1.02–1.29) and a 17% and 16% increased risk of metabolic syndrome (OR: 1.05–1.30 and 1.06–1.32), respectively (Figure 5C).

### 3.6 NWO‐Related Microbial Genera and Modifiable Lifestyles

Overall, we observed consistent results across the WELL‐China and Lanxi cohorts. The genera that were enriched in the NWO group were associated with unhealthier lifestyle factors, such as smoking, drinking and less physical activity, as well as unhealthy dietary patterns characterized by lower intakes of fruit, dairy, seafood and nuts (Figure S2).

## 4 Discussion

In our NWO‐CMD mortality analysis, we found that NWO was associated with a higher risk of CMD mortality in two prospective cohorts. In a subsequent gut microbiota analysis, we found significant gut microbiota alterations in individuals with NWO, and these alterations were significantly associated with CMD in the two independent cohorts. Moreover, we also found that modifiable unhealthy lifestyle factors were correlated with genera that were enriched in the NWO group.

Although obesity is a well‐established risk factor for CMD mortality, limited evidence exists regarding the association between NWO and CMD mortality. To the best of our knowledge, only two studies published in the literature have explored the association between NWO and cardiovascular disease (CVD) mortality, yielding conflicting results. One study involving 1528 older adults found no significant association between NWO and CVD mortality [10], while another study of 3129 women indicated a marginally positive association [11]. This inconsistency may be primarily attributed to insufficient statistical power or number of cases, given the lower CMD mortality rates in people with normal BMI. To provide greater clarity, we conducted a meta‐analysis by combining individual data from the US NHANES and UK Biobank databases. The pooled results showed that NWO was related to a 26% higher risk of CMD mortality. Our analyses included 168 099 adults, more than 30 times the combined sample size of the two studies mentioned. This large sample size provides a more precise estimate and convincing evidence regarding this neglected but important subtype.

In this study, we observed that the prevalence of NWO was 8.37%, 11.33% and 5.67% among the entire population in cohort studies from China, the United States and the United Kingdom, respectively. Overall, these numbers are consistent with those reported in prior publications [6]. Considering the global population and available data regarding the prevalence of NWO, we hypothesized that NWO may impact at least 400 million people worldwide. These data highlight the widespread nature of NWO and its potential impact on global public health.

However, ‘normal weight’ status, as determined by BMI, may act as a barrier to individual's awareness of increased health risks, potentially diminishing their motivation to adopt healthier behaviours [19]. In addition, individuals with NWO often miss out on primary prevention opportunities owing to a lack of routine diagnosis, which in turn leads to a substantial yet neglected disease burden [20].

Similar to NWO, metabolically healthy obese (MHO) is also a specific obesity phenotype. MHO was characterized by individuals who are classified as obese based on their BMI but do not exhibit the typical metabolic abnormalities (TG ≤ 1.7 mmol/L, HDL‐C > 1.0 in men and > 1.3 mmol/L in women, SBP ≤ 130 mmHg, DBP ≤ 85 mmHg, FBP ≤ 5.6 mmol/L and no drug treatment for diabetes and hypertension) [21]. This phenotype challenged the conventional view of obesity, suggesting that not all individuals with obesity are equally at risk for metabolic diseases. A few human studies have shown that microbial genera *Prevotella*, *Prevotellaceae_UCG003* and *Eubacterium rectale* were more prevalent in MHO compared with controls [22]. Yet evidence from large cohort studies on the association between gut microbiota and NWO is particularly lacking.

Our findings revealed that NWO was consistently associated with seven genera in both cohorts. Notably, five of these seven genera including *Fusobacterium*, *R. gnavus group*, *Ruminococcus torques group* (enriched) [23, 24] as well as *Christensenellaceae_R7 _group* and *Coprococcus* (depleted) [25, 26] have previously been linked to obesity. However, the remaining two genera—*Phascolarctobacterium* and *Clostridia_UCG‐014*—have been minimally explored in relation to adult obesity within existing human studies.

To investigate whether *Phascolarctobacterium* and *Clostridia_UCG‐014* exhibit a distinct association with NWO independent of obesity, we further explored their association with overweight and obesity in our study. Our results indicated that no significant associations were observed between these two genera and the overweight/obesity groups (Tables S2 and S3). These findings highlighted the possibility that *Phascolarctobacterium* and *Clostridia_UCG‐014* may represent specific genera associated with NWO. Moreover, we found statistically significant differences in the overall microbial composition between the NWO group and the overweight/obesity group (Figure S3). These novel associations collectively suggested that NWO may have specific microbial signatures, differing from those determined by BMI‐related obesity.

There are similarities and differences between these two genera and the other five genera in metabolite production, metabolic pathways, and lipid and polysaccharide catabolism. *Clostridia_UCG‐014*, *Coprococcus* and *Christensenellaceae_R7_ group* primarily produce the beneficial metabolite butyrate, which is crucial for maintaining gut health, modulating inflammation and supporting gut barrier function [26, 27]. In contrast, *Phascolarctobacterium* primarily produces propionate [27]. *Ruminococcus gnavus* and *Fusobacterium* include pathogenic species that can secrete inflammatory metabolites like polysaccharide A, potentially leading to harmful health outcomes such as inflammatory bowel disease [28, 29]. However, their pathways for metabolite production differ. *Phascolarctobacterium* and *Clostridia_UCG‐014* primarily ferment carbohydrates [30], while *Fusobacterium* is more versatile and capable of metabolizing peptides and amino acids [31]. The *Christensenellaceae_R7_group* and *Coprococcus* similarly ferment dietary fibres and complex polysaccharides, resulting in the production of beneficial SCFAs [26, 32].

Previous studies have demonstrated that lipid metabolism and inflammatory pathways play a role in the development of NWO. Our analyses of microbial function and metabolic pathways not only confirmed the involvement of lipid metabolism (glycerolipid and sphingolipid metabolism) and inflammation (histidine metabolism) but also uncovered new aspects, including amino acid biosynthesis and degradation (valine, leucine and isoleucine) as well as coenzyme A biosynthesis. In vitro, histidine was found to inhibit H_2_O_2_‐ and TNF‐α‐induced IL‐8 secretion in intestinal epithelial cells [33]. In a randomized controlled trial, adding histidine decreased serum levels of inflammatory cytokines (TNF‐α and IL‐6) compared with the placebo group [34]. Leucine and isoleucine, two essential amino acids, are thought to reduce body weight and white adipose tissue (WAT) through the regulation of lipid metabolism‐related genes and the promotion of WAT browning [35]. Moreover, coenzyme A (CoA) is a crucial cofactor in cellular metabolism. Previous animal studies have shown that CoA supplementation reduced adiposity and hepatic lipid accumulation in mice fed high‐fat or high‐carbohydrate diets [36]. Taken together, our findings offer novel insights into the mechanisms of NWO.

To investigate whether the gut microbiota has a similar effect on NWO as inflammation and lipid metabolism, we compared the effect sizes of gut microbiota with those of lipid and inflammatory biomarkers. In the WELL‐China and Lanxi cohorts, the odds ratios (OR) per SD unit increment gut microbiota were 1.18 (95% CI: 1.08–1.27) and 1.23 (95% CI: 1.09–1.37), while the range of ORs for lipid and inflammatory markers was 1.07–1.29 and 1.17–1.25, respectively (Table S4). Overall, the effect size of microbial genera was comparable to those of lipid and inflammatory biomarkers, suggesting that gut microbiota may provide an additional explanation for NWO development.

While NWO has been linked to a higher risk of CMD, the role of NWO‐related gut microbiota in CMD remains unclear. In this study, we addressed this gap and found a statistically significant association between NWO‐related gut microbial features and CMD. Although the underlying mechanisms behind this association warrant further exploration, it is plausible that lipid metabolism and inflammatory pathways play certain roles. Previous research has indicated that *Ruminococcus gnavus* group and *Fusobacterium* induce the secretion of inflammatory cytokines, including tumour necrosis factor‐α (TNF‐α) and interleukin (IL)‐6 [28, 29]. Furthermore, cohort studies have consistently demonstrated a robust association of depleted *Christensenellaceae* with unfavourable lipid profiles, directly contributing to worsened cardiometabolic health [37]. For NWO‐specific genera, the role of *Clostridia_UCG‐014* in metabolic diseases primarily through the production of butyrate. Butyrate enhances insulin receptor function and promotes glucose uptake in peripheral tissues, thereby aiding in blood glucose management [27]. Additionally, it helps maintain blood pressure homeostasis by reducing vascular inflammation and improving endothelial function, which in turn lowers the risk of hypertension [38]. In the case of *Phascolarctobacterium*, studies using high‐fat diet rat models have shown a positive correlation between this bacterium and increased fat mass, which has direct harmful implications for metabolic diseases [39]. Furthermore, it may influence the production of pro‐inflammatory cytokines, contributing to systemic inflammation and worsening metabolic diseases. Despite these findings, further animal models are warranted to determine whether observed association in this study between gut microbiota and CMD risk factors illustrated an actual causal effect.

Modifiable lifestyle factors offer a cost‐effective intervention for reshaping gut microbiome structures [40]. Our study found a consistent correlation between the genera enriched in the NWO group and unhealthy lifestyles, including smoking, drinking and reduced physical activity as well as lower dietary intakes of fruit, dairy, nuts and seafood. Unfavoured lifestyle choices, such as limited exercise and poor dietary habits, have been related to an increased prevalence of NWO and a higher risk of developing of CMD [41]. Based on our findings and existing literature [42], we speculated that lifestyle characteristics are first driving changes in gut microbiota composition. These alterations may subsequently influence fat distribution and CMD risk in individuals with normal BMI. This highlights the importance of healthy lifestyles in terms of improving gut health, even among individuals with normal BMI.

Strengths of our study included consistent observations in two independent cohorts, a large sample size, and the use of DXA for accurate exposure assessments (total fat and fat distribution). However, several limitations of the study should also be acknowledged. First, 16S rRNA gene sequencing technology can only provide accurate genus‐level annotation information of gut microbiota. The current analysis may underestimate the amount of differential gut microbes compared with species‐level classification, but it does not affect our main conclusions. Second, our gut microbiota analysis employed a cross‐sectional design, but our consistent findings for both binary disease and continuous variable analyses in the two independent cohorts and mendelian randomization analysis reinforce the robustness of our results. Third, although we adjusted for a range of covariates, other residual confounders could not be excluded due to the nature of the observational study. As with any observational study, further research using animal models was needed to verify our findings and explore the underlying mechanisms. Such investigations are crucial for developing targeted therapeutic strategies and enhancing our understanding of NWO.

In conclusion, our study observed adiposity‐related gut microbiome alterations among individuals with normal BMI (i.e., NWO). These microbiome alterations are linked to an increased risk of cardiometabolic diseases. Since NWO are frequently overlooked when using BMI as a screening tool for cardiometabolic disease risk, incorporating body fat measurement into routine health screening is crucial for identifying this subgroup in clinical practices.

## Conflicts of Interest

The authors declare no conflicts of interest.

## Supporting information


**Figure S1.** The flow chart of participants selection in the WELL‐China cohort and the Lanxi cohort, respectively
**Figure S2.** NWO‐related gut microbiota and modifiable lifestyles in the WELL‐China cohort (A1, A2) and Lanxi cohort (B1, B2). The Spearman partial correlation analysis was used to calculate the coefficient and *P* value, adjusted for age and sex. Upward arrows indicated the genera enriched in the NWO group, whereas the downward arrows indicated the genera depleted in the NWO group. ***p < 0.001, **0.001 ≤ *p* < 0.01, *0.01 ≤ *p* < 0.05
**Figure S3.** The comparison of gut microbial composition between the NWO and overweight/obesity groups.
**Figure S4.** Diagram of the potential link between habitual lifestyles, gut microbiota, NWO adipose tissue and CMD.
**Table S1.** Mendelian randomization analysis for the association between gut microbiota and cardiometabolic risk indicators
**Table S2.** The association between gut microbial genera and overweight and obesity, with normal‐weight as the reference group in the WELL‐China cohort
**Table S3.** The association between gut microbial genera and overweight and obesity, with normal‐weight as the reference group in the Lanxi cohort
**Table S4.** Association of per standard deviation changes in microbiota score, lipid and inflammatory biomarkers with normal weight obesity (NWO)
**Table S5.** International Classification of Diseases (ICD) codes used for cardiometabolic mortality
