# Supplementary material for: Distinct Gut Microbiota Profiles in Normal Weight Obesity and Their Association With Cardiometabolic Diseases: Results From Two Independent Cohort Studies
Source: J Cachexia Sarcopenia Muscle. 2024 Dec 26;16(1):e13644. doi: 10.1002/jcsm.13644 (PMC11670180; doi:10.1002/jcsm.13644)
Supplement: Supplementary file 1 — Figure S1. The flow chart of participants selection in the WELL‐China cohort and the Lanxi cohort, respectively Figure S2. NWO‐related gut microbiota and modifiable lifestyles in the WELL‐China cohort (A1, A2) and Lanxi cohort (B1, B2). The Spearman partial correlation analysis was used to calculate the coefficient and P value, adjusted for age and sex. Upward arrows indicated the genera enriched in the NWO group, whereas the downward arrows indicated the genera depleted in the NWO group. ***p < 0.001, **0.001 ≤ p < 0.01, *0.01 ≤ p < 0.05 Figure S3. The comparison of gut microbial composition between the NWO and overweight/obesity groups. Figure S4. Diagram of the potential link between habitual lifestyles, gut microbiota, NWO adipose tissue and CMD. Table S1. Mendelian randomization analysis for the association between gut microbiota and cardiometabolic risk indicators Table S2. The association between gut microbial genera and overweight and obesity, with normal‐weight as the reference group in the WELL‐China cohort Table S3. The association between gut microbial genera and overweight and obesity, with normal‐weight as the reference group in the Lanxi cohort Table S4. Association of per standard deviation changes in microbiota score, lipid and inflammatory biomarkers with normal weight obesity (NWO) Table S5. International Classification of Diseases (ICD) codes used for cardiometabolic mortality [file JCSM-16-e13644-s001.docx]

**Supplemental Methods**

**Method S1 Fecal sample collection**

The participants self-collected fecal samples under the guidance of staff, who provided instructions regarding the collection process at the recruitment site. Stool samples were obtained from the participants after defecation, either the night before or the morning of the physical examination day, and separated into two 30-mL cryogenic tubes. The samples were then transported to our laboratory on dry ice within 4 hours, and stored in freezers at -80 ℃ until they were analyzed.

**Method S2 Fecal microbial DNA extraction and 16S rRNA gene sequencing**

Fecal microbial DNA was extracted from each sample using the QIAamp® DNA Stool Mini Kit (Qiagen, Hilden, Germany) according to the manufacturer's instructions. The amplification procedure of 16S ribosomal RNA (rRNA) gene was divided into two PCR steps. In the first PCR reaction, 16S rRNA gene from extracted genomic DNA was amplified using the primes 341F(5’-CCTACGGGNGGCWGCAG-3’) and 805R (5’-GACTACHVGGGTATCTAATCC-3’) directionally targeting the V3 and V4 hypervariable region. Amplification was performed in triplicate 20 μL mixture containing 4 μL FastPfu Buffer, 2 μL dNTPs, 0.1 µM primer 341 F, 0.1 µM primer 805 R and 10 ng of template DNA. Reactions were run in a ABI GeneAmp® 9700 PCR instrument according to the following cycling program: denaturation at 95 °C for 2 min, followed by 25 cycles of 95 °C for 30s (denaturing), 55 °C for 30s (annealing), and 72 °C for 30s (elongation), and a final extension at 72 °C for 5 min. Subsequently, the amplified products were checked by 2% agarose gel electrophoresis and ethidium bromide staining. Amplicons were quantified using a QuantiFluor^TM^-ST quantification system (Promega, USA) according to the manufacturer's instructions. In the second PCR step, sequencing primers and adaptors were added to the ends of the amplicon products to generate indexed libraries ready for downstream sequencing on Illumina platfrom. The concentration of the pooled libraries was determined by the QuantiFluor^TM^-ST quantification system. Finally, DNA libraries were multiplexed and loaded onto the Illumina MiSeq platform (Illumina Inc., CA, USA) according to manufacturer’s instructions. Sequencing was performed using paired-end; image analysis and base calling were conducted by the Control Software embedded in the instrument. Raw paired-end fastq files were processed by using QIIME2 2022.2

**Method S3 16S rRNA gene sequencing bioinformatics**

We used the Quantitative Insights into Microbial Ecology 2 (QIIME 2, 2022.2) platform to perform bioinformatics analysis of gut microbiota.^1^ Raw data were demultiplexed and the imported into QIIME2 platform using the q2-demux followed by denoising with DADA2.^2^ We employed the DADA2 pipeline (q2-dada2) to obtain amplicon sequence variants (ASVs) feature tables and representative sequences by filtering low-quality and duplicated sequences, learning error rates, merging pair reads, and removing primers and chimeras. Based on the classify-sklearn algorithm from q2-feature-classifier plugin, the taxonomic information of each ASVs was annotated against the Silva-138-99 reference database.^3^ All amplicon sequence variants (ASVs) were aligned and used to constructed phylogenetic tree by using mafft (q2‐alignment) and fasttree2 (q2‐phylogeny) respectively. Then, gut microbial α-diversity (observed species, Chao 1 index, Shannon index and Simpson index) and β-diversity (Bray-Curtis distance) parameters was calculated by q2-diversity based on the rarefied ASVs counts (min 20000 sequences). Finally, the genus-level absolute abundance table was extracted from the pipeline and converted to relative abundance table by normalizing to total counts of each genus for comparing the discrepant gut microbiota.

**Method S4 Metabolomic Assessment**

Untargeted metabolomic assays were conducted by Calibra Diagnostics/ Metabolon using plasma samples from participants. The assays utilized Metabolon's HD4 Discovery untargeted metabolomics platform. Sample preparation was performed with the automated MicroLab STAR system® (Hamilton Company, Reno, NV, USA), and analyses were carried out utilizing ultra-performance liquid chromatography-tandem mass spectrometry (UPLC-MS/MS) methods. Detailed information regarding metabolomic assays has been reported previously.

**Method S5 Assessment of dietary and lifestyle factors**

In the WELL-China cohort, habitual dietary data was assessed via a 26-item Food Frequency Questionnaire (FFQ), which was validated and used in previous studies.^4, 5^ In the Lanxi cohort, we used a semi-quantitative FFQ with 58-food items to obtain dietary information, which was designed based on the 2010 China National Nutrition and Health Survey (CNNHS).^6^

Smoking status was classified as never smoker, ever smoker and current smoker. Drinking status was grouped into non-drinker, occasional drinker (others), and frequent drinker (≥12 times per year). Physical activity was categorized into the inactive group, insufficiently active group, and active group based on the short version of the International Physical Activity Questionnaire.^7^ Pittsburgh Sleep Quality Index (PSQI) was used to assess sleep quality, the PSQI score ≤ 7 and >7 was defined as good sleep quality and poor sleep quality, respectively.^8^ According to a previous study, we used television watching as the proxy for recreational sedentary behaviour, and sedentary was defined as television watching ≥ 4 hour/day.^9^

**Methods S6 Definition of normal weight obesity (NWO) in the US NHANES and the UK Biobank**

In US NHANES and UK Biobank, normal weight obesity (NWO) was defined as participants with normal BMI (18.5-24.9 kg/m^2^) but excess percent body fat (≥ 25% in men and ≥ 35% in women). Whole-body DXA scans (Hologic, Inc.; Bedford, MA, USA) and Tanita BC418MA body composition analyzer (Tanita, Tokyo, Japan) were used to measure the participants’ percent body fat, respectively. Normal weight lean (NWL) was defined as participants with normal BMI and normal percent body fat (< 25% in men and < 35% in women).

**Methods S7 Definition of cardiometabolic mortality in the US NHANES and the UK Biobank**

In US NAHNES, mortality data was obtained by linkage to the National Death Index through 31 December 2019. In UK Biobank, mortality data were obtained by linkage to national death registries through 30 September 2021 (England and Wales) or 31 October 2021 (Scotland). For cardiometabolic mortality, we combined deaths classified as diseases of heart, cerebrovascular diseases, essential hypertension and diabetes mellitus. The International Classification of Diseases, Tenth revision (ICD-10) was used to determine cardiometabolic mortality in both cohorts (**Table S5**).

**Supplementary Table 1** Mendelian randomization analysis for the association between gut microbiota and cardiometabolic risk indicators

| **CMD risk indicators** | **No. of SNP** | **OR (95%CIs)** | ***P*-value** |
| --- | --- | --- | --- |
|  | ***Fusobacterium*** | | |
| Glucose | 7 | 1.01(0.97-1.06) | 0.646 |
| **SBP** | **12** | **1.78(1.05-3.00)** | **0.031** |
| **DBP** | **12** | **1.44(1.06-1.97)** | **0.021** |
| HDL-C | 18 | 0.98(0.96-1.01) | 0.256 |
| LDL-C | 18 | 1.01(0.98-1.04) | 0.328 |
| TG | 18 | 1.00(0.97-1.03) | 0.900 |
| TC | 7 | 1.01(0.97-1.05) | 0.554 |
| CRP | 17 | 1.01(0.98-1.04) | 0.632 |
| **Uric acid** | **13** | **1.08(1.01-1.16)** | **0.030** |
|  | ***Ruminococcus torques*** | | |
| Glucose | 5 | 1.00(0.96-1.03) | 0.999 |
| SBP | 9 | 0.66(0.26-1.66) | 0.378 |
| DBP | 9 | 0.64(0.39-1.05) | 0.078 |
| HDL-C | 9 | 1.01(0.98-1.05) | 0.409 |
| LDL-C | 9 | 0.93(0.83-1.04) | 0.234 |
| TG | 9 | 0.94(0.87-1.03) | 0.086 |
| TC | 5 | 0.89(0.72-1.11) | 0.325 |
| CRP | 9 | 0.94(0.87-1.02) | 0.093 |
| Uric acid | 9 | 0.97(0.91-1.03) | 0.325 |
|  | ***Ruminococcus gnavus*** | | |
| Glucose | 4 | 0.98(0.96-1.01) | 0.118 |
| SBP | 12 | 1.09(0.75-1.59) | 0.659 |
| DBP | 12 | 1.11(0.89-1.39) | 0.347 |
| HDL-C | 12 | 0.99(0.98-1.01) | 0.659 |
| LDL-C | 12 | 1.00(0.98-1.02) | 0.812 |
| TG | 12 | 1.00(0.98-1.02) | 0.927 |
| TC | 4 | 0.99(0.97-1.02) | 0.594 |
| CRP | 12 | 0.99(0.97-1.01) | 0.264 |
| Uric acid | 12 | 0.99(0.97-1.03) | 0.760 |
|  | ***Phascolarctobacterium*** | | |
| Glucose | 9 | 1.02(0.99-1.04) | 0.160 |
| SBP | 8 | 0.88(0.46-1.70) | 0.720 |
| DBP | 8 | 0.85(0.70-1.02) | 0.081 |
| HDL-C | 8 | 1.00(0.98-1.02) | 0.901 |
| LDL-C | 8 | 0.98(0.95-1.02) | 0.318 |
| TG | 8 | 0.99(0.97-1.01) | 0.527 |
| TC | 9 | 0.98(0.96-1.01) | 0.200 |
| CRP | 9 | 0.99(0.97-1.01) | 0.375 |
| Uric acid | 9 | 0.97(0.92-1.02) | 0.182 |
|  | ***Christensenellaceae_R7_group*** | | |
| Glucose | 10 | 0.98(0.95-1.02) | 0.382 |
| SBP | 9 | 1.09(0.67-1.79) | 0.721 |
| DBP | 9 | 1.19(0.95-1.49) | 0.131 |
| HDL-C | **10** | **1.04(1.00-1.08)** | **0.034** |
| LDL-C | 10 | 0.99(0.96-1.03) | 0.711 |
| TG | 10 | 0.99(0.94-1.04) | 0.776 |
| TC | 4 | 0.99(0.89-1.10) | 0.843 |
| CRP | 10 | 0.99(0.96-1.03) | 0.711 |
| Uric acid | 10 | 1.00(0.96-1.05) | 0.850 |
|  | ***Coprococcus*** | | |
| Glucose | 11 | 0.99(0.97-1.01) | 0.406 |
| SBP | 10 | 1.02(0.67-1.55) | 0.911 |
| DBP | 11 | 0.93(0.77-1.13) | 0.475 |
| HDL-C | **10** | **1.02(1.00-1.05)** | **0.038** |
| LDL-C | 11 | 1.02(0.99-1.04) | 0.219 |
| TG | 11 | 0.98(0.96-1.02) | 0.382 |
| TC | 6 | 0.98(0.91-1.06) | 0.597 |
| CRP | **9** | **0.97(0.95-0.99)** | **0.010** |
| Uric acid | 12 | 0.98(0.96-1.01) | 0.232 |

SNP column represents the number of SNPs used in data analyses

**Table S2** The association between gut microbial genera and overweight and obesity, with normal-weight as the reference group in the WELL-China cohort

| **Genus** | **Coefficient** | ***P* value** | **Q value^c^** |
| --- | --- | --- | --- |
| **Overweight** | | | |
| *UCG-002* | -0.462 | 0.000 | 0.000 |
| ***Christensenellaceae_R7_group*** | -0.393 | 0.000 | 0.000 |
| *Lachnospiraceae_NK4A136_group* | -0.285 | 0.000 | 0.000 |
| *Alistipes* | -0.285 | 0.000 | 0.000 |
| *Akkermansia* | -0.283 | 0.001 | 0.003 |
| *Megamonas* | 0.265 | 0.001 | 0.004 |
| ***Ruminococcus_torques_group*** | 0.150 | 0.001 | 0.005 |
| *Romboutsia* | 0.193 | 0.002 | 0.007 |
| *Lachnoclostridium* | 0.139 | 0.003 | 0.012 |
| ***Fusobacterium*** | 0.216 | 0.005 | 0.020 |
| *EscherichiaShigella* | 0.155 | 0.012 | 0.039 |
| *Eubacterium_coprostanoligenes_group* | -0.158 | 0.012 | 0.039 |
| *Monoglobus* | -0.137 | 0.015 | 0.044 |
| *Bacteroides* | -0.092 | 0.017 | 0.048 |
| *Clostridium_sensu_stricto_1* | 0.146 | 0.025 | 0.067 |
| *Lactobacillus* | 0.181 | 0.031 | 0.078 |
| ***Ruminococcus_gnavus_group*** | 0.137 | 0.031 | 0.078 |
| *Faecalibacterium* | -0.087 | 0.041 | 0.097 |
| *Ruminococcus* | -0.114 | 0.051 | 0.115 |
| *Parasutterella* | 0.146 | 0.052 | 0.116 |
| *g__Subdoligranulum* | -0.101 | 0.078 | 0.157 |
| *g__Klebsiella* | 0.125 | 0.084 | 0.167 |
| *g__Blautia* | 0.067 | 0.102 | 0.195 |
| *Other* | 0.055 | 0.113 | 0.210 |
| **Obesity** | | | |
| *Romboutsia* | 0.591 | 0.000 | 0.000 |
| *Dorea* | 0.416 | 0.000 | 0.000 |
| *Megamonas* | 0.626 | 0.000 | 0.000 |
| *Bacteroides* | -0.297 | 0.000 | 0.000 |
| *Clostridium_sensu_stricto_1* | 0.448 | 0.000 | 0.000 |
| *Alistipes* | -0.412 | 0.000 | 0.000 |
| *UCG-002* | -0.422 | 0.000 | 0.000 |
| ***Ruminococcus_torques_group*** | 0.282 | 0.000 | 0.001 |
| *Agathobacter* | 0.317 | 0.000 | 0.001 |
| *Lachnospiraceae_NK4A136_group* | -0.316 | 0.000 | 0.001 |
| ***Christensenellaceae_R7_group*** | -0.405 | 0.000 | 0.002 |
| *Streptococcus* | 0.318 | 0.001 | 0.003 |
| *Collinsella* | 0.351 | 0.001 | 0.005 |
| *Akkermansia* | -0.411 | 0.002 | 0.008 |
| *Monoglobus* | -0.269 | 0.003 | 0.011 |
| *Parabacteroides* | -0.233 | 0.003 | 0.014 |
| ***Coprococcus*** | 0.255 | 0.006 | 0.021 |
| *Prevotella* | 0.326 | 0.006 | 0.023 |
| *Lactobacillus* | 0.331 | 0.013 | 0.041 |
| *Ruminococcus* | -0.229 | 0.014 | 0.043 |
| ***Fusobacterium*** | 0.284 | 0.020 | 0.057 |
| *Lachnoclostridium* | 0.167 | 0.024 | 0.065 |
| *Bifidobacterium* | -0.232 | 0.026 | 0.068 |
| *Blautia* | 0.141 | 0.031 | 0.078 |
| *Eubacterium_hallii_group* | 0.176 | 0.051 | 0.115 |
| *Dialister* | 0.225 | 0.059 | 0.126 |
| *EscherichiaShigella* | 0.179 | 0.069 | 0.142 |

**Table S3** The association between gut microbial genera and overweight and obesity, with normal-weight as the reference group in the Lanxi cohort

| **Genus** | **Coefficient** | ***P* value** | **Q value^c^** |
| --- | --- | --- | --- |
| **Overweight** | | | |
| *Lachnospiraceae_NK4A136_group* | -0.293 | 0.0001 | 0.001 |
| *EscherichiaShigella* | 0.239 | 0.002 | 0.016 |
| *Megamonas* | 0.312 | 0.004 | 0.021 |
| *UCG_002* | -0.275 | 0.004 | 0.021 |
| *Roseburia* | -0.189 | 0.005 | 0.027 |
| *Romboutsia* | 0.219 | 0.009 | 0.041 |
| *Bacteroides* | -0.130 | 0.010 | 0.046 |
| ***Fusobacterium*** | 0.278 | 0.011 | 0.049 |
| *Dorea* | 0.168 | 0.015 | 0.062 |
| ***Christensenellaceae_R7_group*** | -0.234 | 0.018 | 0.071 |
| *Parabacteroides* | -0.165 | 0.018 | 0.072 |
| *Clostridium_sensu_stricto_1* | 0.207 | 0.020 | 0.076 |
| *Alistipes* | -0.187 | 0.035 | 0.115 |
| *Barnesiella* | -0.235 | 0.038 | 0.120 |
| *Collinsella* | 0.1791 | 0.040 | 0.125 |
| *Subdoligranulum* | -0.136 | 0.093 | 0.240 |
| *Enterobacter* | 0.167 | 0.09 | 0.242 |
| **Obesity** | | | |
| *Megamonas* | 1.001 | 4.56E-08 | 8.18E-07 |
| *Lachnospiraceae_NK4A136_group* | -0.708 | 8.58E-08 | 1.45E-06 |
| ***Fusobacterium*** | 0.988 | 9.92E-08 | 1.59E-06 |
| ***Christensenellaceae_R7_group*** | -0.697 | 2.78E-05 | 0.0003 |
| *UCG_002* | -0.651 | 5.23E-05 | 0.0005 |
| *Romboutsia* | 0.524 | 0.0002 | 0.001 |
| *Bacteroides* | -0.288 | 0.0007 | 0.004 |
| *Dorea* | 0.385 | 0.001 | 0.006 |
| ***Ruminococcus_gnavus_group*** | 0.487 | 0.001 | 0.007 |
| *Parabacteroides* | -0.344 | 0.003 | 0.019 |
| *EscherichiaShigella* | 0.384 | 0.004 | 0.022 |
| *Barnesiella* | -0.520 | 0.006 | 0.030 |
| ***Ruminococcus_torques_group*** | 0.290 | 0.009 | 0.044 |
| *Faecalibacterium* | -0.250 | 0.015 | 0.062 |
| *Clostridium_sensu_stricto_1* | 0.354 | 0.018 | 0.072 |
| *Alistipes* | -0.348 | 0.019 | 0.074 |
| ***Coprococcus*** | -0.454 | 0.021 | 0.077 |
| *Collinsella* | 0.296 | 0.042 | 0.132 |
| *Lachnoclostridium* | 0.221 | 0.050 | 0.152 |
| *Monoglobus* | -0.235 | 0.078 | 0.209 |

**Table S4** Association of per standard deviation changes in microbiota score, lipid and inflammatory biomarkers with normal weight obesity (NWO)

| **Biomarkers** | **Odds ratio** | **95%CIs** | ***P*-value** |
| --- | --- | --- | --- |
|  | WELL-China cohort | | |
| NWO-related microbiota index | 1.18 | 1.08-1.27 | <0.001 |
| LDL-cholesterol | 1.29 | 1.20-1.41 | <0.001 |
| Triglyceride | 1.13 | 1.05-1.22 | 0.001 |
| Total cholesterol | 1.25 | 1.15-1.36 | <0.001 |
| C-reactive protein | 1.07 | 0.99-1.15 | 0.069 |
| White cell count | 1.25 | 1.14-1.37 | <0.001 |
|  | Lanxi cohort | | |
| NWO-related microbiota index | 1.23 | 1.09-1.37 | <0.001 |
| LDL-cholesterol | 1.21 | 1.09-1.35 | <0.001 |
| Triglyceride | 1.19 | 1.08-1.32 | 0.001 |
| Total cholesterol | 1.17 | 1.06-1.31 | 0.003 |
| C-reactive protein | 1.23 | 1.09-1.38 | 0.001 |
| White cell count | 1.25 | 1.12-1.39 | <0.001 |

Biomarker concentrations were natural log-transformed and standardized by z-score.

Model adjusted for age, sex, marriage, smoking, drinking, physical activity, education, income, total energy intake, and antibiotic use.

**Table S5** International Classification of Diseases (ICD) codes used for cardiometabolic mortality

| **Cause of death** | **ICD 10** | |
| --- | --- | --- |
|  | US NHANES | UK Biobank |
| Diseases of heart | I00-I09, I11, I13, I20-I51 | I00-I09, I11, I13, I20-I51 |
| Cerebrovascular diseases | I60-I69 | I60-I69 |
| Diabetes mellitus | E10-E14 | E10-E14 |
| Essential hypertension and hypertensive renal disease | — | I10, I12 |


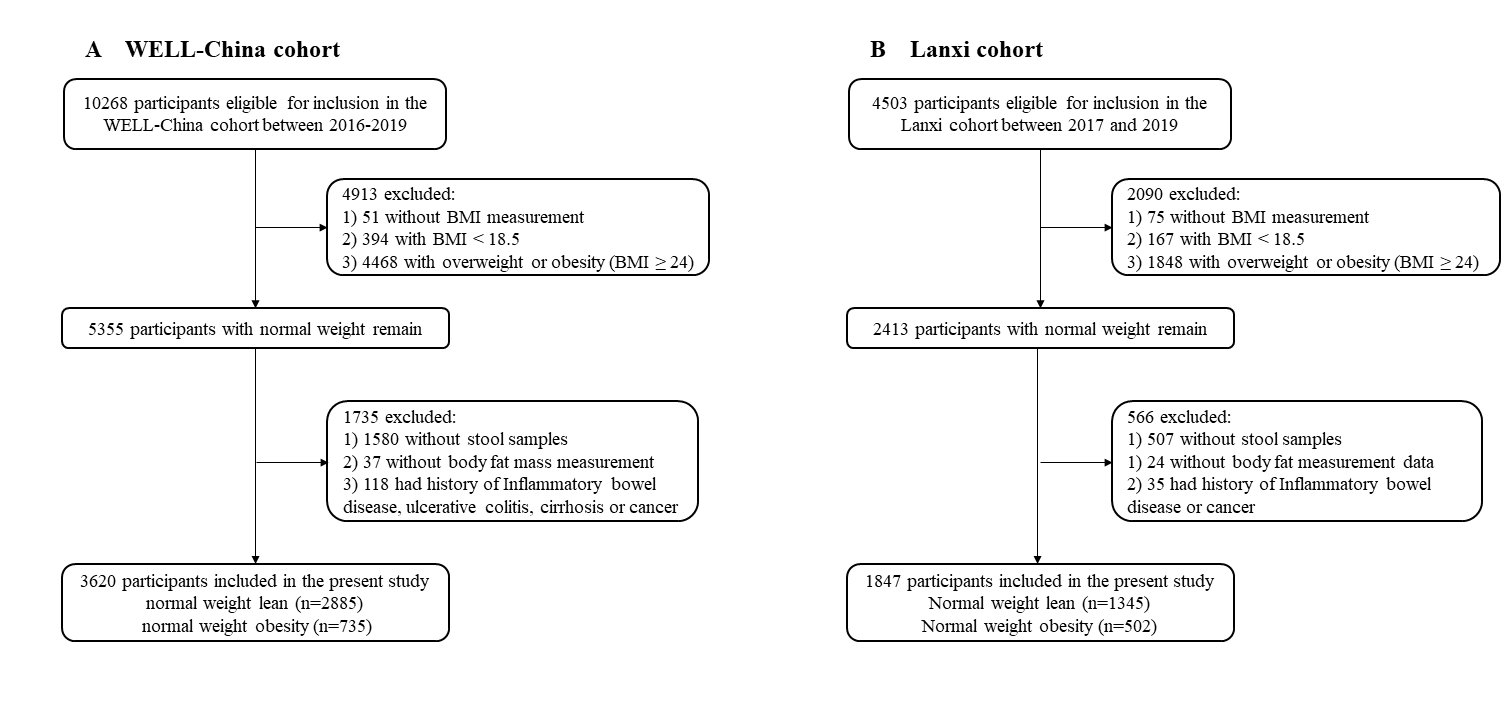


**Figure S1** The flow chart of participants selection in the WELL-China cohort and the Lanxi cohort, respectively


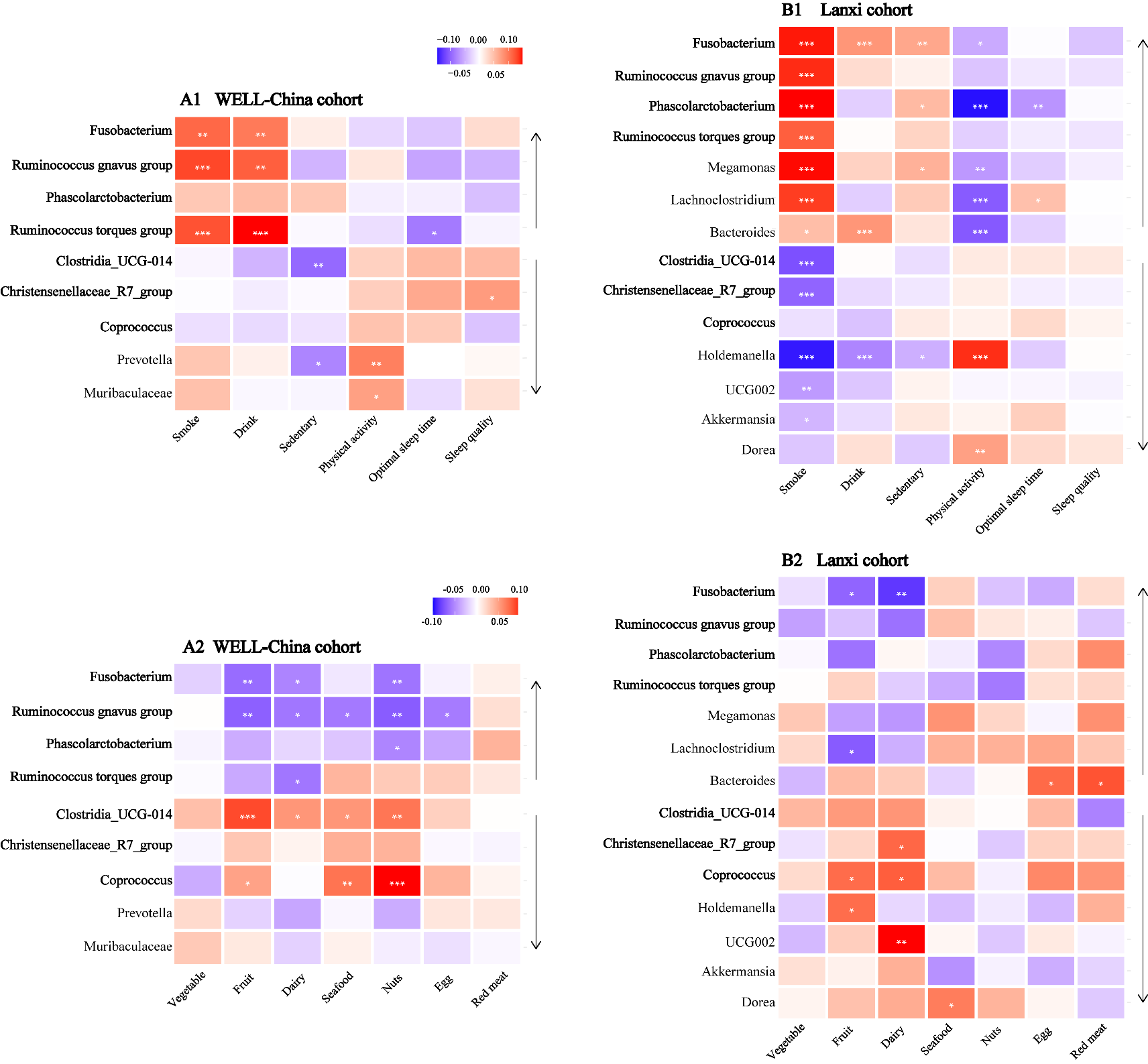
**Figure S2 NWO-related gut microbiota and modifiable lifestyles in the WELL-China cohort (A1, A2) and Lanxi cohort (B1, B2).** The Spearman partial correlation analysis was used to calculate the coefficient and *P* value, adjusted for age and sex. Upward arrows indicated the genera enriched in the NWO group, whereas the downward arrows indicated the genera depleted in the NWO group. ****P*<0.001, **0.001≤*P*<0.01, *0.01≤*P*<0.05


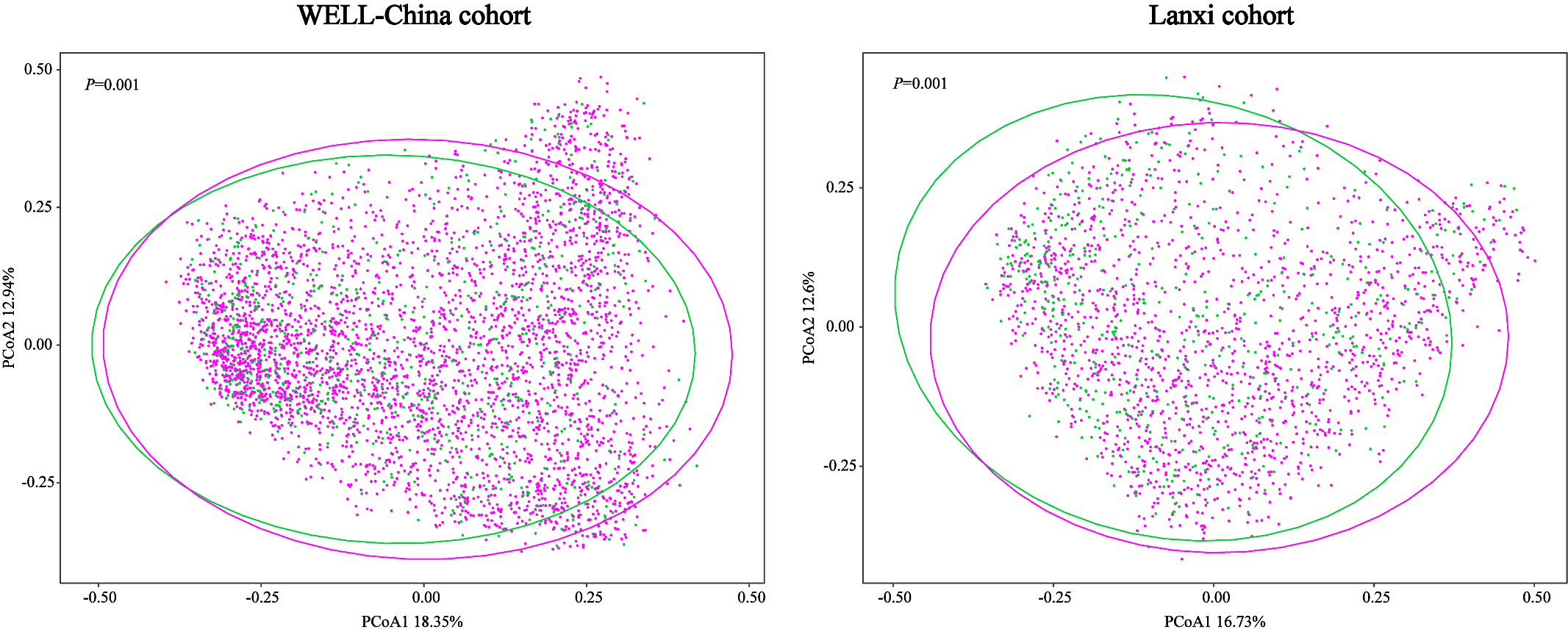


**Figure S3** The comparison of gut microbial composition between the NWO and overweight/obesity groups.

**
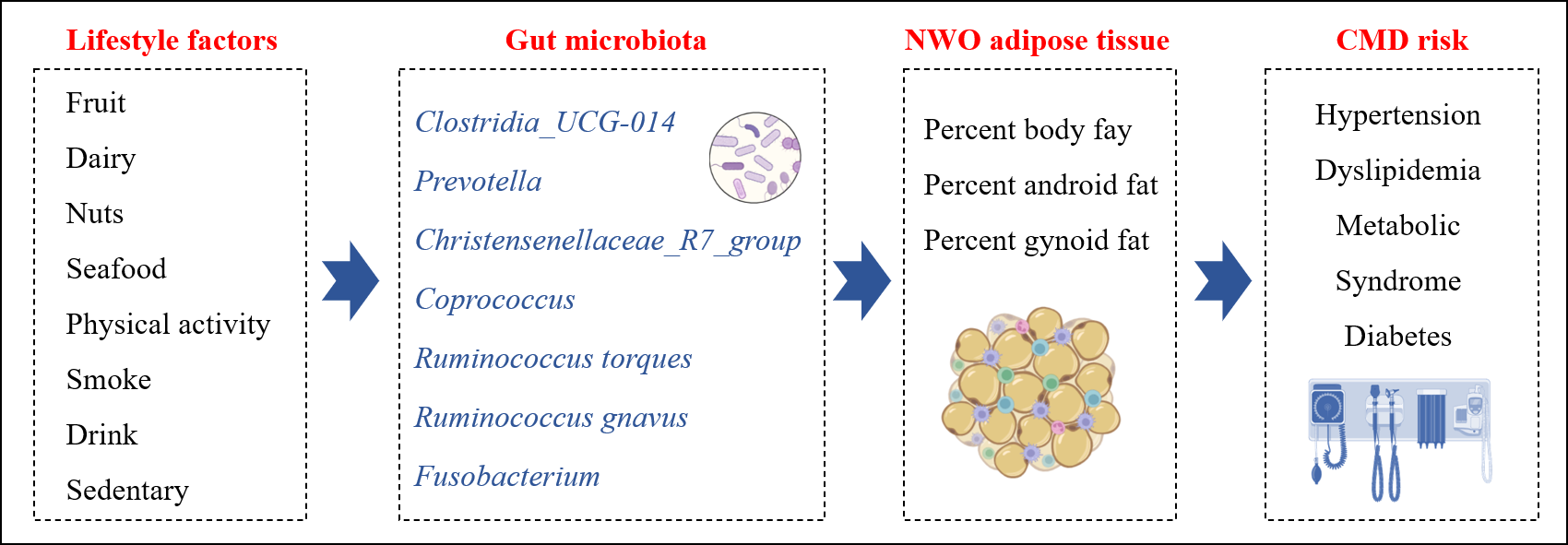
**

**Figure S4** Diagram of the potential link between habitual lifestyles, gut microbiota, NWO adipose tissue, and CMD.

**References**

1. Bolyen E*, et al.* Reproducible, interactive, scalable and extensible microbiome data science using QIIME 2. *Nat Biotechnol* **37**, 852-857 (2019).

2. Callahan BJ, McMurdie PJ, Rosen MJ, Han AW, Johnson AJ, Holmes SP. DADA2: High-resolution sample inference from Illumina amplicon data. *Nat Methods* **13**, 581-583 (2016).

3. Bokulich NA*, et al.* Optimizing taxonomic classification of marker-gene amplicon sequences with QIIME 2's q2-feature-classifier plugin. *Microbiome* **6**, 90 (2018).

4. Ru Y*, et al.* Characterization of dietary patterns and assessment of their relationships with metabolomic profiles: A community-based study. *Clin Nutr* **40**, 3531-3541 (2021).

5. Gao J. Association of dietary patterns and physical activities with total body fat proportions and metabolic syndrome among middle-aged and elderly people: a cross-sectional study. *Department of Nutrition and Food Hygiene*, (2012).

6. Shu L*, et al.* Association between Dietary Patterns and the Indicators of Obesity among Chinese: A Cross-Sectional Study. *Nutrients* **7**, 7995-8009 (2015).

7. Fan M, Lyu J, He P. [Chinese guidelines for data processing and analysis concerning the International Physical Activity Questionnaire]. *Zhonghua Liu Xing Bing Xue Za Zhi* **35**, 961-964 (2014).

8. Spira AP*, et al.* Reliability and validity of the Pittsburgh Sleep Quality Index and the Epworth Sleepiness Scale in older men. *J Gerontol A Biol Sci Med Sci* **67**, 433-439 (2012).

9. van de Vegte YJ, Said MA, Rienstra M, van der Harst P, Verweij N. Genome-wide association studies and Mendelian randomization analyses for leisure sedentary behaviours. *Nat Commun* **11**, 1770 (2020).
